# Typhoid Fever in Relation to Privies, Water-Closets, and Rateable Value

**Published:** 1898-11-05

**Authors:** 


### TYPHOID FEVER IN RELATION TO PRIVIES, WATER-CLOSETS, AND RATEABLE VALUE.

Dr. Charles Porter, the medical officer of health for Stockport, who has done much valuable work in tracing out the relations of typhoid fever to circumstances other than water supply, draws attention to some curious statistics which he has collected in regard to the varying incidence of this disease on different classes of house in Stockport during the past five years. He has tabulated the houses as privy houses and water-closet houses, and has also arranged both classes in groups according to their rateable value. It was, perhaps, to be expected that in all cases the prevalence of typhoid should be greater among the privy houses. At any rate, such is shown to be the fact. Thus, not only do we see that midden privy towns are more liable than water-closet towns to the occurrence of typhoid fever, but that in a town of mixed methods this disease occurs with far greater frequency among the privy houses than among those provided with water-closets. Of course, the retort is ready to hand that the privy houses are the houses of the poor, and the others the houses of the well-to-do, and that, therefore, there is nothing to be surprised at in this distribution of the disease. It is here, however, that Dr. Porter's thoroughness and carefulness comes in, for he has taken the trouble to sort out each set of cases according to the rateable value of the houses, with the result of showing that, taking houses of the same rateable value, it is always the case that the disease falls most heavily upon the privy houses; thus proving that it is the existence close at hand of the filthy midden-privy, which acts as a perfect incubator for the typhoid bacillus, that is the cause of the typhoid prevalence, and not the social position of the inhabitants of the houses. This matter, indeed, is clenched by the still more surprising result of Dr. Porter's investigation, namely, that in each class the disease is proportionately more prevalent in the better houses. This is a matter, which is by no means easy of explanation, but it is one of the most interesting facts brought out by Dr. Porter's laborious investigations. Many practical men have long thought that the closed doors, the tightly-fitting windows, and the general superiority of the joinery of better class houses, with the resulting lack of ventilation, are by no means conducive to health, and that there is still much to be said for the old-fashioned, breezy upper chamber, which ventilates through the roof, and for the perpetual open door so characteristic of the living room of the poorer cottage, and it certainly is a matter of considerable interest to find Dr. Porter saying that the figures for Stockport show that the incidence of typhoid fever increases with rateable value, both in privy-pit and water-closet houses. In these latter days we seek for comfort above all things —close, warm, stuffy comfort. But comfort is by no means synonymous with health.

